# Small Molecules Targeting Kidney ClC-K Chloride Channels: Applications in Rare Tubulopathies and Common Cardiovascular Diseases

**DOI:** 10.3390/biom13040710

**Published:** 2023-04-21

**Authors:** Maria Antonietta Coppola, Michael Pusch, Paola Imbrici, Antonella Liantonio

**Affiliations:** 1 Department of Pharmacy–Drug Sciences, University of Bari “Aldo Moro”, 70125 Bari, Italy; maria.coppola@uniba.it; 2 Institute of Biophysics, National Research Council, 16149 Genova, Italy; michael.pusch@ibf.cnr.it

**Keywords:** ClC-K, kidney, Bartter syndrome, hypertension

## Abstract

Given the key role played by ClC-K chloride channels in kidney and inner ear physiology and pathology, they can be considered important targets for drug discovery. Indeed, ClC-Ka and ClC-Kb inhibition would interfere with the urine countercurrent concentration mechanism in Henle’s loop, which is responsible for the reabsorption of water and electrolytes from the collecting duct, producing a diuretic and antihypertensive effect. On the other hand, ClC-K/barttin channel dysfunctions in Bartter Syndrome with or without deafness will require the pharmacological recovery of channel expression and/or activity. In these cases, a channel activator or chaperone would be appealing. Starting from a brief description of the physio-pathological role of ClC-K channels in renal function, this review aims to provide an overview of the recent progress in the discovery of ClC-K channel modulators.

## 1. Introduction

Human ClC-Ka and ClC-Kb are double-barreled chloride channels belonging to the family of ClC proteins [1,2,3,4]. ClC-Ka and ClC-Kb are homologous epithelial chloride channels mainly expressed in the kidney and inner ear, where they play a pivotal role in chloride homeostasis.

Over the last few years, pharmacological research has been committed to developing drugs acting as modulators of ClC-K channels with the aim of providing better therapeutic treatment for patients affected by ClC-K-associated disorders. The identification of barttin as an essential ClC-K subunit [5,6,7] and the resolution of the 3D structure of the first mammalian ClC-K [8] allowed significant advances in the biophysical and pharmacological characterization of ClC-K channels, establishing a mechanistic model for chloride transport and for ligand binding.

## 2. Role of ClC-K Channels in Health and Disease

**Figure 1:**
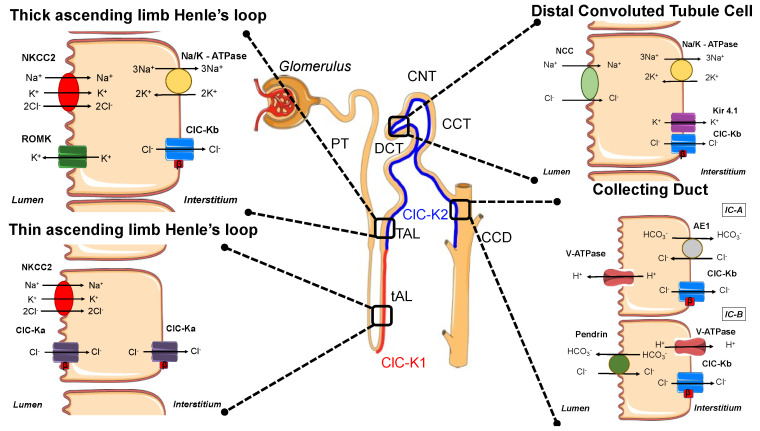
Overview of ClC-K chloride channels’ distribution at distinct nephron sites. Pattern of expression of rat orthologues: ClC-K1 (red line) in the thin ascending limb (tAL) and ClC-K2 (blue line) in thick ascending limb (TAL) through distal convoluted tubule (DCT) and in intercalated cells (IC-A and IC-B) of collecting duct (CD). ClC-Ka (dark violet) mainly expressed in tAL in both apical and basolateral membrane. ClC-Kb (blue) expression in the basolateral membrane along the nephron participating in Cl^−^ reabsorption. Both isoforms are represented with their accessory subunit β barttin (red). Loss of function of chloride channels causes Bartter Syndrome type III. NKCC2, Na^+^K^+^2Cl^−^ cotransporter; ROMK, renal outer medullary potassium channel.

Human *CLCNKA* and *CLCNKB* genes encoding the human ClC-Ka and ClC-Kb channel proteins, respectively, are located very close to each other on human chromosome 1p36.13 [9]. The two chloride channels are named ClC-K to highlight their predominant expression in the kidney [4]. Even though the two isoforms have 90% sequence identity at the protein level, their expression pattern and specific roles are different along the nephron (Figure 1; Table 1) [9]. Additionally, their single-channel conductance is different [10,11]. An important breakthrough in the study of ClC-K proteins was the discovery of barttin, their accessory β-subunit. In 2001, Estevez and co-authors demonstrated, through immunohistochemistry experiments, the co-expression of both ClC-K channels and barttin in the basolateral membranes of the marginal cells of the vascular stria [6], evidence that was confirmed later for the kidney channels as well. The accessory β-subunit barttin (encoded by the *BSND* gene) is necessary for ClC-Ks’ expression at the plasma membrane and gating [7]. 

**Figure 2:**
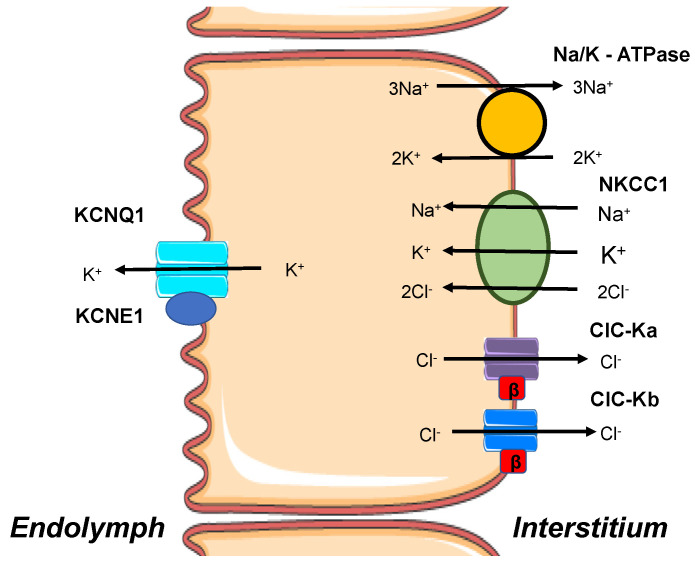
Chloride channels’ expression in the inner ear. In strial marginal cells, basolateral ClC-Ka (dark violet) and ClC-Kb (blue) together with their auxiliary subunit β-barttin (red) recycle Cl^−^. Loss of barttin or loss of both isoforms of chloride channels causes deafness (Bartter Syndrome type IV).

Loss-of-function mutations in the *CLCNKB* gene cause Bartter Syndrome (BS) type III, a rare tubulopathy characterized by hypokalemia, metabolic alkalosis, polyuria, and increased renin and aldosterone levels, with normal or low blood pressure [12,13,14]. The two isoforms are also expressed in the inner ear (Figure 2), where they are important for endolymph secretion and consequently for the proper function of hearing [6,9,15]. A more severe form with similar renal symptoms to BS type III, called Bartter Syndrome type IVa, is characterized by additional deafness [16]. BS type IVa is caused by mutations in the *BSND* gene that encodes the accessory subunit barttin [17]. Since both ClC-Ka and ClC-Kb rely on barttin for proper membrane expression and function, the loss of barttin function impacts both ClC-K homologues, explaining the more severe phenotype of BS IVa compared to BS III. Similarly, the loss-of-function of both genes encoding ClC-Ka and ClC-Kb is associated with Bartter Syndrome type IVb [18,19]. Currently, the long-term treatment of BS consists of a high-salt diet, potassium and magnesium supplement, and potassium-sparing diuretics, such as spironolactone/eplerenone and amiloride, to normalize electrolyte balance. Such a symptomatic therapeutic approach is associated with a series of side effects, and several clinical disputes regarding the safety of the current management of BS exist [20]. Thus, a switch from a symptomatic to a defect-targeted approach is needed to allow a more specific and safer therapy. 

Considering that ClC-Kb and ClC-Ka are important players in the renal tubular chloride reabsorption mechanism [21,22,23,24] and the cross-talk between the kidney and heart, mutations or polymorphisms causing increased or decreased activity in these channels may be associated with cardiovascular diseases. Importantly, renal dysfunction is common in heart failure and hypertension and is associated with an increased risk of mortality, supporting ClC-K channels as appealing therapeutic targets in this context. Indeed, polymorphisms that alterate ClC-K channels’ activity seem to be associated with hypertension and heart failure [25,26,27,28]. This finding requires confirmation in larger cohorts of patients [29,30,31,32] Particularly, hypertensive patients presenting the A447T or Y351F polymorphism within the *CLCNKA* gene showed an increase in blood pressure in response to an acute Na-load, indicating *CLCNKA* as a candidate gene in salt-sensitive hypertensive subjects [25]. Another study suggests that the polymorphism R83G in the *CLCNKA* gene represents a common risk allele for heart failure [26]. In particular, the R83G variant exhibited reduced chloride currents compared to wild-type ClC-Ka when expressed in tsA201 cells [26]. It has been speculated that hyperreninemia may occur in response to this ClC-Ka loss-of-function variant, leading to an increased risk of heart failure [26,33]. With respect to ClC-Kb, Jeck et al. reported that a common *CLCNKB* polymorphism (T481S) led to a seven-fold larger current amplitude compared to wild-type ClC-Kb when expressed in *Xenopus* oocytes [27]. This gain of function correlated with higher blood pressure and decreased glomerular filtration rate in tested individuals [27,34]. Importantly, ethnic factors seem to impact on the association between the T481S polymorphism and the predisposition to essential hypertension. Indeed, *CLCNKB* T481S is associated with essential hypertension in males within the Ghanaian population, while the same polymorphism seems not to play a role in BP regulation in a Swedish cohort [32,35]. Further recent studies supported the role of ClC-K channels within the cardio–renal axis. Indeed, the use of animal models allowed the revelation of the key role played by ClC-K channels in the etiopathogenesis of hypertension and heart failure, through their involvement into molecular mechanisms associated with inflammatory pathways [11,36,37,38]. 

**Table 1:** Localization and physiological role of ClC-K channels in the kidney and inner ear as assessed from animal models.

Protein	Kidney Localization	Kidney Function	Mouse Models	References
ClC-K1/ClC-Ka	Apical and basolateral membrane of tAL at internal medulla. Additionally, at TAL and CD at internal medulla	Passive transepithelial reabsorption of Cl^−^ in tAL that creates a cortico-medullary osmotic gradient that favors the reabsorption of water from the collecting duct	KO mice showed diabetes insipidus	[24]
ClC-K2/ClC-Kb	Basolateral membrane of TAL, DCT, connecting tubule and collective tubule of external cortex and medulla	Secondary active NaCl reabsorption and urine concentration; maintenance of the transepithelial potential that determines the paracellular absorption of Ca^2+^ and Mg^2+^	KO mice showed Bartter-like symptoms: salt loss, marked hypokalemia, and metabolic alkalosis	[11,37,38,39]
barttin	Basolateral membranes of the marginal cells of the stria vascularis, co-expressed with ClC-Ks in the kidney	Generation of the endocochlear potential and transepithelial Cl^−^ transport	KO mice died within a few days after birth due to severe salt loss and dehydration and had congenital deafness. R8L KI mice showed Bartter-like symptoms with reduced transepithelial chloride transport	[40,41]

tAL, thin ascending limb of Henle’s loop; TAL, thick ascending limb of Henle’s loop; DCT, distal convoluted tubule; CD, collecting duct.

## 3. Structure–Function Relationship in ClC-K Channels

Pioneering studies of CLC proteins from bacteria and eukaryotes greatly contributed to the understanding of the main structural features and biophysical properties of CLC proteins [9]. CLC proteins present a homodimeric structure (double-pore) with anion binding sites at the center of each subunit [42,43,44,45]. The single subunit is composed of 18 alpha helices (from A to R), 17 of which are partially embedded in the membrane. The pore is not formed at the interface between subunits, but each subunit forms its own independent pore [9]. The negatively charged side chain of E148 located in helix F (with numbering referring to the *Escherichia coli* (ecClC) homologue) [46], known as the “gating glutamate”, is responsible for the voltage-dependent gating observed in CLC proteins functioning as chloride channels, such as ClC-1, the CLC isoform selectively expressed in skeletal muscle [9]. The intracellular C-terminus of eukaryotic CLC channels harbors two tandem cystathionine-beta-synthase (CBS) domains. The cytoplasmic domains are likely involved in the modulation of the not-well understood mechanism of “common gating”, a gating process that simultaneously opens and closes both pores of the double-barreled channel [9,47,48]. In ClC-1, the CBS domains appear to be relevant for expression, slow gating and intracellular modulation [49,50,51,52,53,54,55,56]. 

In 2006 and 2007, Markovic and colleagues crystallized the CBS domains from the *Torpedo marmorata* channel ClC-0, human ClC-Ka, and human ClC-5 [52,53,57]. The main feature discovered was that the structure of the CBS domain in ClC-K, a chloride channel distantly related to ClC-0 and ClC-5 transporters, shared same oligomeric organization, which is unique for this protein family. Indeed, the dimeric structure of the cytoplasmic domains of the ClC-Ka at 1.6 Å resolution revealed a strong structural similarity with a cytoplasmic portion of ClC-5 and ClC-0 [57]. Interestingly, while ATP was found to be bound in the ClC-5 CBS domains, no nucleotides were present in the structures of the ClC-Ka and ClC-0 domains [57]. It remains to be studied whether the cytoplasmic domains of ClC-K channels have functional roles for conformational changes or are involved in intracellular responses to the binding of unknown ligands.

**Figure 3:**
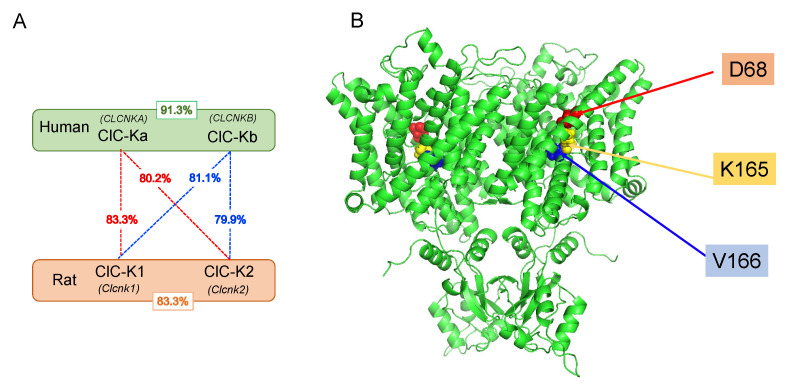
Structure and sequence homology of ClC-K channels. (**A**) The picture shows the sequence homology within ClC-Ks channels in the human and the rat orthologue. (**B**) Three-dimensional representation of ClC-Kb modeled upon cryo-electron microscopy structure of the bovin ClC-K (PDB id: 5TQQ; [8]) showing the localization of the most important residues for ClC-K channels: V166 (blue spheres), D68 (red spheres) and K165 (yellow spheres).

Recently, the structure of the bovine ClC-K has been resolved [8] (note that bovine has only one ClC-K gene). The structure of the ion permeation pathway described by Park et al. is generally similar to that of all other CLC proteins, such as, for example, the red algal Cyanidioschyzon merolae (cmClC) and the bacterial ecClC [42,58]. In particular, previous studies showed the presence of three consecutive anion binding sites at the center of each subunit, which are named external (Sext), central (Scent) and internal (Sint) sites, underlining the position of each site through the pore [42,58,59,60]. Even though the bovine ClC-K shares a homodimeric double-pore structure with algal and bacterial CLC proteins, the ClC-K structure reveals important details about the interactions of some residues which surround Sext and Scent sites in the pore region [8]. In particular, differences in the conformation of a highly conserved serine residue in the intracellular pore entrance close to Scent and Sint could explain differences between the anion–proton exchange function of ecClC and cmClC compared to the channel behavior of ClC-K. One principal difference between ClC-K channels and most other CLCs regards the gating glutamate, which is replaced by valine 166 in human ClC-Ka and ClC-Kb (Figure 3). The absence of a negatively charged side-chain renders ClC-K channels voltage independent [6,61,62,63]. In the bovine ClC-K structure, the side chain of V166 is pointed parallel to the channel wall [8]. Furthermore, the cytosolic vestibule of ClC-K was positively charged, which could lead to a local increase in the concentration of Cl^−^ ions [8]. The organization of the CBS domains in the ClC-K structure was similar to that described for cmClC, but their orientation relative to the transmembrane part was rotated by ~20 degrees compared with the cmClC structure [8,58]. Louet and co-authors built a homology model of the main part of human ClC-Kb using the 3D structures of the bovine ClC-K, the eukaryotic cmClC and the bacterial ecClC structures [8,46,58,64]. Interestingly, in the pore region of the human model there was an additional salt-bridge between residues D68 and K165, compared to the bovine structure (Figure 3) [64]. In this respect, and as described in more detail below, the role of D68 to determine ClC-K sensitivity to blockers is noteworthy [65]. Despite being only a homology model, the high quality of the structures it is built on provides confidence that the model could be used as a tool for further structural studies as well as in silico drug discovery investigations [64]. 

As said before, similar to other ClC members [66,67], ClC-K channels associate with barttin [5,6,68,69]. For ClC-7, the structure of the complex formed with its β subunit Ostm1 has been determined [70,71]. However, so far, no structure has been reported of the ClC-K/barttin complex. This remains an important goal for a better understanding of the stoichiometry and the interactions between the two subunits, which might be of considerable pharmacological relevance. 

## 4. Discovery of ClC-K Chloride Channel Modulators

**Figure 4:**
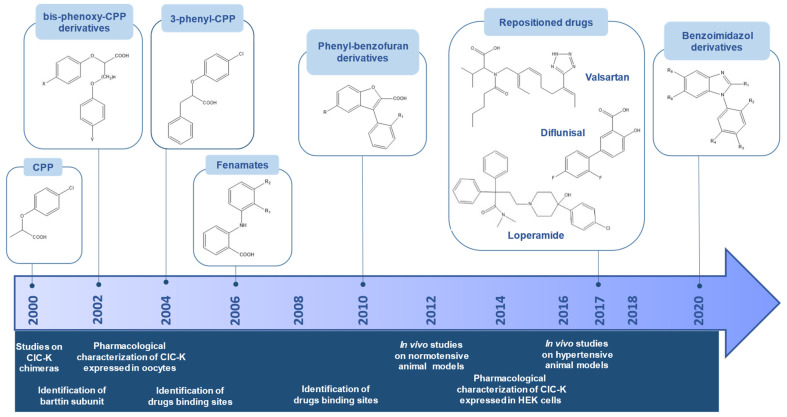
Picture describing the discovery of ClC-K ligands in the last 20 years.

Figure 4 depicts the discovery of ClC-K ligands in the last 20 years, as detailed in the following sections. 

### 4.1. 2-p-Chlorophenoxypropionic Acid (CPP) and Derivatives

Before the discovery of barttin [6], only murine ClC-K1 could be functionally expressed in heterologous systems [4]. However, the expression level was too low to allow a detailed pharmacological characterization. To overcome these limits, Waldegger and Jentsch created chimeras between murine ClC-K1 and human ClC-Kb that were successively used for preliminary pharmacological investigations [61,72]. In these studies, various derivatives of 2-p-chlorophenoxypropionic acid (CPP) were tested as potential inhibitors of ClC-K channels. CPP was considered a potential starting lead compound since it was an efficient blocker of the native skeletal muscle chloride conductance [73] as well as of heterologously expressed ClC-1 [74,75]. Interestingly, bis-phenoxy derivatives of CPP were able to block chimeric ClC-K channels when applied from the extracellular side (IC_50_ ~100 μM) (Table 2) [72]. Among CPP derivatives, GF-100 was active on wild type ClC-K1 co-expressed with barttin with an apparent K_D_ of about 100 μM [72]. GF-100 was also effective on ClC-1, but only if applied from the intracellular side, while it showed almost no effect on ClC-K1 from the intracellular side [72]. Successively, by starting from GF-100, a wide structure–activity relationship (SAR) study was performed with the aim of identifying the simplest structure among GF-100 derivatives that was able to inhibit ClC-K channels with high potency. Importantly, 3-phenyl-CPP was finally identified as the most potent inhibitor, able to block ClC-K1 from the extracellular side with an IC_50_ of ~100 μM. The block was voltage-dependent and increased by reducing the extracellular chloride concentration, suggesting the competition of the blocker with chloride ions in the channel pore [76]. Furthermore, by screening various classical Cl-channel blockers, it was also found that NFA (niflumic acid) and DIDS (4,4′-Diisothiocyano-2,2′-stilbenedisulfonic acid) were capable of blocking ClC-K1 with K_D_ values in the micromolar range (Table 2) [65]. 

The discovery of barttin allowed the pharmacological characterization of the human ClC-Ka and ClC-Kb homologues. Interestingly, 3-phenyl-CPP blocked ClC-Ka with IC_50_ < 100 μM, whereas ClC-Kb was less sensitive to this compound. ClC-Ka was also blocked by DIDS with an IC_50_ of about 90 μM, and even in this case the block of ClC-Ka was stronger than block of ClC-Kb. Both compounds blocked more efficiently at positive voltages [65].

The different pharmacological sensitivity of ClC-Ka compared to ClC-Kb could later be attributed to two residues located in helix B in the extracellular vestibule: asparagine 68 and glycine 72 of ClC-Ka were replaced by aspartate 68 and glutamate 72 in ClC-Kb, respectively [65]. When the neutral residues in ClC-Ka were replaced by the negatively charged ClC-Kb residues, the block of ClC-Ka was as low as in ClC-Kb. Probably, the negatively charged residues created unfavorable electrostatic interactions with the negative charges on the blocker structures [65]. The most crucial position for binding was found to be 68. It has been speculated that position 72, if negatively charged, creates additional repulsive electrostatic forces which, interacting with the second negative charge of the DIDS, prevent the binding and render ClC-Kb less sensitive to blockage [65]. Importantly, in aqueous solutions DIDS is unstable and hydrolysis products can be formed [77]. The five major isolated products were distinguished into five fractions and named as follows, 4,4′-diaminostilbene-2,2′-disulfonic acid (DADS), dimer, trimer, tetramer and pentamer, as a consequence of DIDS and DADS units’ oligomerization. These resulting compounds were able to bind ClC-Ka, revealing them to be the most powerful inhibitors known for ClC-Ka (IC_50_ < 2 μM) [78]. Despite being valuable pharmacological tools, DIDS derivatives cannot be used in clinics because of the lack of specificity and their chemical instability.

**Table 2:** Drug effects on ClC-K channels.

Drug	Molecular Structure	P/NP	ClC-K Isoform	Effect on Current	Kd (μM)	Expression System	Reference
Bis-phenoxy CPP,	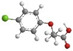	NP	ClC-K1	↓	~110	*Xenopus* Oocytes	[72]
3-phenyl CPP and			ClC-Ka	↓	~80	*Xenopus* Oocytes	[65]
derivatives			ClC-Kb	↓	~400	*Xenopus* Oocytes	[65]
GF-166	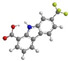	P	ClC-Ka	↑	n.d	*Xenopus* Oocytes	[79]
			ClC-K1	↓	~150	*Xenopus* Oocytes	[76]
DIDS	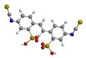	NP	ClC-Ka	↓	90	*Xenopus* Oocytes	[65]
			ClC-Kb	↓	~580	*Xenopus* Oocytes	[65]
	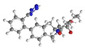		ClC-K1	↓	~50	HEK293 cells	[80]
Valsartan		NP	ClC-Ka	↓	21	HEK293 cells	[80]
			ClC-Kb	↓	~100	HEK293 cells	[80]
Indomethacin	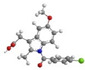	NP	ClC-Ka	↓	400	*Xenopus* Oocytes	[81]
RT-93	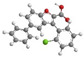	NP	ClC-K1	↓	~10	*Xenopus* Oocytes	[33]
			ClC-Ka	↓	~11	HEK293 cells	[82]
			ClC-Kb	↓	~10	*Xenopus* Oocytes	[79]
				↓	~15	HEK293 cells	[82]
SRA-36	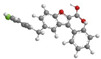		ClC-K1	↓	~6	HEK293 cells	[83]
		NP	ClC-Ka	↓	~6	HEK293 cells	[83]
			ClC-Kb	↓	~6	HEK293 cells	[83]
Diflunisal	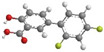	NP	ClC-Kb	↓	15	*Xenopus* Oocytes	[32]
Loperamide	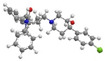	NP	ClC-Kb	↓	34	*Xenopus* Oocytes	[32]
BIM	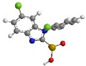	NP	ClC-Ka	↓	~10	*Xenopus* Oocytes	[84]
			ClC-Kb	↓	200	*Xenopus* Oocytes	[84]

P, planar; NP, not planar; ↓, reduction; ↑, increase.

### 4.2. Niflumic Acid and Other Fenamates

Further studies in Xenopus oocytes revealed that micromolar concentrations of NFA augmented ClC-Ka- and ClC-Kb-mediated anionic currents (Table 3). In particular, NFA had a biphasic effect on ClC-Ka: at low concentrations (<1 mM) it caused an enhancement on currents, while high NFA concentrations (≥1 mM) blocked the currents. In contrast, ClC-Kb currents were augmented at all concentrations to a maximum of about four-fold [85]. The biphasic effect of NFA on ClC-Ka suggested the presence of two NFA binding sites with different affinities. According to the proposed model, the opening of the channel is associated with a high affinity binding site, while the block of the channel is determined by the binding of NFA to a lower affinity site. A mutagenesis study attempted to identify the putative NFA activating binding sites on ClC-Ka channels [86]. Three key residues could be identified (L155, G345, A349), localized in two different protein regions that, based on the crystal structure of homologous bacterial ClC-1, are expected to be exposed to the extracellular side of the channel, relatively close to each other. In 2014, Pusch and his group identified two adjacent amino acids, F256 and N257, at the beginning of the I-J loop that, when mutated, hugely altered the biphasic response of ClC-Ka to NFA, with F256A being potentiated 37-fold and N257A being potently blocked with an IC_50_ ~1 μM [87]. These residues are localized in the same extracellular I-J loop, which harbors a regulatory Ca^2+^ binding site [87]. After these initial reports, the biophysical and pharmacological behaviors of human ClC-K channels expressed either in HEK293 cells or in *Xenopus* oocytes have been compared. It was demonstrated that the NFA-mediated current potentiation found for ClC-Ks expressed in oocytes [85] was not observed in HEK293 and MDCK cells, where NFA blocked both ClC-Ka and ClC-Kb at all tested concentrations [82]. One hypothesis to explain the different response to NFA may be that the activating binding site is masked and less easily accessible when human ClC-Ks are expressed in HEK293 cells. Conversely, flufenamic acid (FFA), whose structure is very similar to NFA, only caused a ClC-Ka channel block, even in oocytes (Table 3) [85]. Interestingly, FFA derivatives did not inhibit ClC-Kb and the blocker-insensitive ClC-Ka mutant N68D, indicating that the activating binding site was distinct from the blocker site. The notable difference between NFA (activation) and FFA (block) effects on ClC-Ka channels could be explained by a fundamental structural difference between these two molecules: NFA has a rigid structure, in which aromatic rings are co-planar; in FFA, the nitrogen atom is absent on the intermediate aromatic ring, producing a steric obstruction which alters co-planarity [85]. Similarly, 3-phenyl-CPP also has a non-co-planar, flexible conformation. This hypothesis [85] was confirmed through the design of non-co-planar derivatives, flexible compared to NFA, and of co-planar derivatives, rigid compared to FFA [79]. Triflocin differs from NFA for the position of nitrogen pyridine, which causes a distortion of the structure, causing the molecule to be non-co-planar, similar to what was observed for FFA. According to the previous hypothesis, triflocin is able to induce the block of ClC-Ka at all tested concentrations [79]. On the other hand, the co-planar derivative of FFA, GF-166, in which the two phenyl groups are forced to co-planarity by a cyclization, was able to activate ClC-Ka at concentrations lower than 200 μM (Table 2) [79]. Thus, the evaluation of the sensitivity of ClC-Ka to the derivatives of NFA and FFA, together with a modeling study of these ligands, allowed the conclusion that one major characteristic of activating compounds is the coplanarity of the two rings of the molecules, whereas the block requires a non-co-planar configuration. The major molecular determinant that distinguishes activators from blockers is the level of planarity of the aromatic portions of the molecules; only molecules with perfectly co-planar aromatic groups display potentiating activity.

In parallel with these studies, Picollo et al. demonstrated that the COX inhibitor non-co-planar indomethacin blocks ClC-Ka channels expressed in *Xenopus* oocytes with an IC_50_ of 400 μM (Table 2) [81]. 

### 4.3. Optimization of the Bis-Phenoxy Carboxylic Acids inhibitors: The Phenyl-Benzofuran Derivatives 

Based on the above-mentioned considerations, combining several molecular features of various ClC-K ligands, it was discovered that phenyl-benzofuran carboxylic acid derivatives yield the most potent ClC-Ka inhibitors so far described (IC_50_ < 10 μM). Indeed, non-coplanar carboxylic acids phenyl-benzofuran derivatives of CPP (MT-189 and RT-93) were identified as specific ligands able to block ClC-K channels’ activity with IC_50_ values less than 10 μM (Table 2). The higher affinity benzofuran derivative RT-93 was identified as the most potent blocker of heterologously expressed ClC-Kb channels, with an IC_50_ value of ~10 μM [79]. Importantly, acute in vivo administration of such newly synthesized molecules resulted in significant diuretic and antihypertensive effects in normotensive rats [88]. Successively, another series of benzofuran derivatives was developed with the aim of identifying molecules with enhanced affinity. The efficacy of these compounds was tested on ClC-K channels expressed in HEK293 cells. Starting from RT-93 and MT-189, it was discovered that SRA-36 is the most potent ClC-K blocker, able to inhibit chloride currents sustained by ClC-Ka and ClC-Kb (Table 2) [83].

Importantly, this molecule was also able to block two ClC-Ka polymorphisms putatively associated with hypertension, such as A447T and Y315F. Furthermore, acute in vivo administration of SRA-36 to spontaneously hypertensive rats caused a significant reduction in blood pressure, confirming the hypothesis that ClC-K channel inhibitors could represent promising drugs to treat hypertension [83]. 

### 4.4. Valsartan and Other AT1 Receptor Antagonists 

Recently, to identify novel ClC-K ligands among prompt-to-use marketed drugs, an innovative drug discovery strategy was used which was based on the search of a pharmacovigilance database monitoring drug safety [80]. In particular, the FDA pharmacovigilance database was searched for commercial drugs that induce a Bartter-like syndrome as a side effect. The assumption was that BS could be causatively related to the blocking of ClC-K channels. From the analysis of the pharmacovigilance registry, the authors identified several commercial drugs and validated their ability to bind and block ClC-K channels through an integrated experimental and computational approach. Patch clamp electrophysiology in HEK293 cells was used to test the effect of antihypertensive AT1 receptor antagonists, sartans, on ClC-K channels. It was found that valsartan, at a concentration of 50 μM, was able to block ClC-Ka/barttin by 55% (Table 2). Conversely, ClC-Kb/barttin channels are less sensitive because only with concentrations of ≥ 100 μM was an inhibition of 40% reached (Table 2) [80]. To determine the structural components of valsartan responsible for the inhibition of ClC-Ka channels and to verify their specificity, other molecules belonging to the same class were tested, such as losartan, telmisartan, candesartan and olmesartan. Losartan, candesartan-cilexetyl and telmisartan, at a concentration of 50 μM, were mild ClC-Ka/barttin blockers reducing currents by 10–25%. 

SAR studies allowed the hypothesis that AT1R antagonists which had a tetrazole ring and a carboxylic group, such as valsartan and olmesartan (IC_50_ = 25 μM and 100 μM, respectively), could effectively block ClC-Ka/barttin [80]. Molecular docking studies showed that the carboxylic group and the tetrazole ring of valsartan and olmesartan interacted with the positively charged K165 residue and with the N68 residue of ClC-Ka. Since ClC-Kb has an aspartic acid at position residue, this ClC-K isoform shows a lower affinity for valsartan (Table 2) [80]. All these findings highlight the importance of the interactions between carboxyl and the tetrazole group of sartans with specific ClC-Ka residues and the greater selectivity and binding affinity of valsartan and olmesartan towards ClC-Ka compared to losartan [80]. This information is crucial from the perspective of using the identified drugs as chaperons and/or leads for the discovery of alternative marked drugs as high affinity ClC-Ks ligands potentially useful in BS.

### 4.5. Diflusinal and Loperamide

Based on the recently published bovine ClC-K structure [8], Louet and collaborators explored, through a receptor-based virtual screening approach, a subset of FDA drugs, limiting the choice to those molecules that could be used orally [64]. For this in silico drug screening, a homology model of human ClC-Kb based on the bovine ClC-K structure was used and RT-93 was considered a reference compound. A total of 1125 FDA compounds was docked in a cavity which was part of the extracellular pore vestibule comprising, e.g., the above-mentioned D68 and E72 residues. Interestingly, the authors performed a docking study that predicted this cavity as suitable to be the most likely small molecule ligand binding site, compared to other accessible cavities present on other proteins [64]. Among the tested compounds, six chemically different molecules inhibited ClC-Kb currents by at least 40%, including the non-steroidal anti-inflammatory drug diflunisal and the long-acting anti-diarrheal drug loperamide (Table 2). Moreover, Louet et al. confirmed data obtained with RT-93, since drug screening did not reveal any substance with higher affinity than RT-93, and, at the same time, strongly supported that the chemical structures of identified drugs, together with RT-93, could be used to design novel ClC-Kb channel inhibitors.

### 4.6. N-Arylated Benzimidazole Derivatives (BIMs)

Finally, to identify more selective ligands of ClC-Ka/Kb channels, Koster and collaborators recently synthetized a new collection of N-arylated benzimidazole derivatives (BIMs), produced with analogous molecular topology of benzofuran derivatives [83,84]. One of the new tested molecules, BIM1, at 100 μM on *Xenopus* oocytes, showed an efficacious inhibition of ClC-Ka but a markedly reduced inhibition of ClC-Kb (>20-fold selectivity for ClC-Ka over ClC-Kb) (Table 2). The authors also performed computational docking to a ClC-Ka homology model identifying the BIM1 binding site on the extracellular face of the protein, near residue N68, in the same region discussed above [65,85,89]. The residue N68 was one of only ∼20 extracellular residues that differed between ClC-Ka and ClC-Kb. Like CPP derivatives [65], the mutation of this residue in ClC-Ka and ClC-Kb (N68D and D68N, respectively) reversed the preference of BIM1 for ClC-Ka over ClC-Kb, thus showing the critical role of residue 68 in establishing BIM1 selectivity [84], very similar to the results of Picollo et al. [65]. Using two electrode voltage-clamp, a series of BIM derivatives were tested on ClC-Ka and ClC-Kb currents to give insight into the increased selectivity of BIM1 compared with other inhibitors. BIM15 and BIM16 were found to be slightly more potent than BIM1, inhibiting ClC-Ka by 54 and 72%, respectively, at 5 μM compared with 37% for BIM1, but this effect was accompanied by a loss in selectivity. The same authors also identified the fundamental structural characteristics necessary for inhibition. The more polar BIM scaffold (BIM1) disfavors the more hydrophobic ClC-Kb binding site compared with the analogous benzofuran derivative (MT-189) and inhibitors bearing large hydrophobic substituents (BIM4 and BIM5 as well as BIM14 to BIM16) [84]. These last studies will aid efforts to develop ClC-Ka inhibitors with increased potency and ClC-K isoform selectivity.

### 4.7. Physiological Modulation of ClC-K Activity: The Role of Calcium Ions and pH

Physiologically relevant modulators of ClC-K channels can be extracellular Ca^2+^ and H^+^. Extracellular Ca^2+^ and H^+^ were reported to regulate the activity of ClC-K channels in vitro and in situ [2,5,6,61,90], with a high sensitivity to Ca^2+^ and H^+^ at physiological concentrations [89]. Importantly, recordings of endogenous chloride channels in kidney tubules, with properties reminiscent of ClC-K channels, revealed that Ca^2+^ and H^+^ act as gating modifiers that influence the open probability with no impact on single channel conductance [89]. 

A detailed dose–response analysis suggested the presence of distinct binding sites for Ca^2+^ and H^+^ [89]. To determine Ca^2+^ and H^+^ regulatory sites, Gradogna et al. performed a mutagenesis screening using the known crystal structure of the distantly related ecClC as a scaffold, substituting all charged and titratable residues on the extracellular side by neutral or non-titratable residues [89]. Two putative residues involved in Ca^2+^ binding, E261 and D278 belonging to the I-J loop, were identified. Interestingly, the two symmetrically located Ca^2+^ binding sites were formed at the outer edge of the interface of the two subunits, with E261 and D278 belonging to different subunits at each site. The contribution of D278 to Ca^2+^ binding was less than that of E261. E261 was not conserved in other eukaryotic channel CLCs, which is probably the reason for the unique Ca^2+^ sensitivity of ClC-K channels [91]. Further studies guided by homology modeling led to the identification of E259 and E281 residues as additionally contributing to Ca^2+^ modulation [91]. A similar approach has been applied to determine the inhibitory H^+^ binding site. A promising candidate residue was H497, because the homologous residue in ClC-2 had been shown to mediate a pH-induced block of the channel [92,93]. In fact, mutagenesis confirmed the importance of H497 for the acid-induced inhibition of ClC-K channels [89]. A change in sensitivity to Ca^2+^ and pH has been reported for a ClC-Kb variant associated with BS III [94], highlighting the importance of this kind of physiological modulation. Furthermore, it has to be remembered that all CLC family members display pH sensitivity [9,46,86,93,95,96,97,98], but only the two ClC-K isoforms are influenced by extracellular [Ca^2+^]. 

The identification of the Ca^2+^ and H+ binding sites, likely ATP binding sites [99], could be useful for the design of compounds directed towards involved residues with pharmacological relevance [100].

## 5. Conclusions

Based on the involvement of ClC-Ka and ClC-Kb channels in Bartter syndrome and in hypertension, there is a great interest in the identification of ClC-Ks channels’ modulators. On one hand, pharmacological research aims to identify selective ClC-K openers to counteract the loss of function defect caused by Bartter syndrome mutations. To date, niflumic acid is the only known activator of ClC-K channels expressed in *Xenopus* oocytes, despite losing this ability when channels are expressed in mammalian cells. On the other hand, specific ClC-K blockers would be useful to control salt and fluid balance. In particular, specific ClC-Ka channel modulators could regulate aqueous diuresis without altering osmotic diuresis [65]. The increase of aqueous diuresis could be beneficial for reducing cardiac load, for example in the treatment of congestive heart failure [100] or in the treatment of hypertensive states characterized by edema [25,27]. Like renal transporters, which represent a target for known molecules with diuretic and antihypertensive activity, ClC-K channels could also represent a cellular target for drugs with high therapeutic potential for cardiovascular diseases [100].

## Figures and Tables

**Table 3:** Summary of the effects of fenamates on chloride currents sustained by ClC-K channels.

Drug	Molecular Structure	P/NP	ClC-K Isoform	Effect on Current	[Drug μM] Range Tested	Expression System	Reference
			ClC-K1	↓	from 1 to 1000	*Xenopus* oocytes	[81]
				↓	from 1 to 1000	HEK293 cells	[82]
			ClC-Ka	↑	from 50 to 1000	*Xenopus* oocytes	[81,85]
	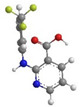			↓	>1000	*Xenopus* oocytes	[81,85]
Niflumic acid		P		↓	from 1 to 1000	HEK293 and MDCK cells	[82]
			ClC-Kb	↑	from 50 to 1000	*Xenopus* Oocytes	[85]
				↓	from 1 to 1000	HEK293 and MDCK cells	[82]
Flufenamic acid	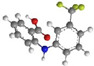	NP	ClC-Ka	↓	from 1 to 2000	*Xenopus* Oocytes	[85]
			ClC-Kb	↑	200	*Xenopus* Oocytes	[85]

P, planar; NP, not planar; ↓, reduction; ↑, increase.

## Data Availability

Data available in a publicly accessible repository.
